# eNODAL: an experimentally guided nutriomics data clustering method to unravel complex drug–diet interactions

**DOI:** 10.1093/bib/bbaf036

**Published:** 2025-02-21

**Authors:** Xiangnan Xu, Alistair M Senior, David G Le Couteur, Victoria C Cogger, David Raubenheimer, David E James, Benjamin Parker, Stephen J Simpson, Samuel Muller, Jean Y H Yang

**Affiliations:** Chair of Statistics, Humboldt-Universität zu Berlin, Unter den Linden 6, Berlin 10178, Germany; Charles Perkins Centre, University of Sydney, Johns Hopkins Drive, NSW 2050, Australia; Sydney Precision Data Science Centre, University of Sydney, F07 Eastern Avenue, NSW 2050, Australia; Laboratory of Data Discovery for Health Limited (D24H), 19 Science Park W Avenue, Hong Kong SAR 999077, China; Charles Perkins Centre, University of Sydney, Johns Hopkins Drive, NSW 2050, Australia; Centre for Education and Research on Ageing, Concord RG Hospital, Hospital Road, NSW 2138, Australia; ANZAC Research Institute, Concord RG Hospital, Hospital Road, NSW 2138, Australia; Centre for Education and Research on Ageing, Concord RG Hospital, Hospital Road, NSW 2138, Australia; ANZAC Research Institute, Concord RG Hospital, Hospital Road, NSW 2138, Australia; Charles Perkins Centre, University of Sydney, Johns Hopkins Drive, NSW 2050, Australia; School of Life and Environmental Science, University of Sydney, F22 Eastern Avenue, NSW 2050, Australia; Charles Perkins Centre, University of Sydney, Johns Hopkins Drive, NSW 2050, Australia; ANZAC Research Institute, Concord RG Hospital, Hospital Road, NSW 2138, Australia; Department of Anatomy and Physiology, University of Melbourne, 30 Royal Parade, VIC 3052, Australia; Charles Perkins Centre, University of Sydney, Johns Hopkins Drive, NSW 2050, Australia; School of Life and Environmental Science, University of Sydney, F22 Eastern Avenue, NSW 2050, Australia; Sydney Precision Data Science Centre, University of Sydney, F07 Eastern Avenue, NSW 2050, Australia; School of Mathematical and Physical Sciences, Macquarie University, 18 Wally's Walk, NSW 2109, Australia; School of Mathematics and Statistics, University of Sydney, F07 Eastern Avenue, NSW 2050, Australia; Charles Perkins Centre, University of Sydney, Johns Hopkins Drive, NSW 2050, Australia; Sydney Precision Data Science Centre, University of Sydney, F07 Eastern Avenue, NSW 2050, Australia; Laboratory of Data Discovery for Health Limited (D24H), 19 Science Park W Avenue, Hong Kong SAR 999077, China; School of Mathematics and Statistics, University of Sydney, F07 Eastern Avenue, NSW 2050, Australia

**Keywords:** nutriomics, drug–diet interaction, interpretable clustering, nonparametric ANOVA

## Abstract

Unraveling the complex interplay between nutrients and drugs via their effects on “omics” features could revolutionize our fundamental understanding of nutritional physiology, personalized nutrition, and, ultimately, human health span. Experimental studies in nutrition are starting to use large-scale “omics” experiments to pick apart the effects of such interacting factors. However, the high dimensionality of the omics features, coupled with complex fully factorial experimental designs, poses a challenge to the analysis. Current strategies for analyzing such types of data are based on between-feature correlations. However, these techniques risk overlooking important signals that arise from the experimental design and produce clusters that are hard to interpret. We present a novel approach for analyzing high-dimensional outcomes in nutriomics experiments, termed experiment-guided NutriOmics DatA cLustering (‘eNODAL’). This three-step hybrid framework takes advantage of both Analysis of Variance (ANOVA)-type analyses and unsupervised learning methods to extract maximum information from experimental nutriomics studies. First, eNODAL categorizes the omics features into interpretable groups based on the significance of response to the different experimental variables using an ANOVA-like test. Such groups may include the main effects of a nutritional intervention and drug exposure or their interaction. Second, consensus clustering is performed within each interpretable group to further identify subclusters of features with similar response profiles to these experimental factors. Third, eNODAL annotates these subclusters based on their experimental responses and biological pathways enriched within the subcluster. We validate eNODAL using data from a mouse experiment to test for the interaction effects of macronutrient intake and drugs that target aging mechanisms in mice.

## Introduction

Nutrition is a powerful determinant of health and disease, but disentangling the single and interactive influences of nutrients and other dietary constituents poses considerable challenges, which are overlooked in conventional one-nutrient-at-a-time approaches [1, 2]. Adding to this complexity is the fact that nutritional requirements differ with genotype, development, infection, and other environmental circumstances [3]. Diet may also interact with non-nutritional factors such as drug treatments [4]. Understanding how nutrients interact with one another and with such external factors to affect multiple levels of physiology and health is at the forefront of nutriomics, precision medicine, and public health.

Preclinical nutrition science is now equipped with conceptual frameworks and multifactorial experimental designs [5], such as the geometric framework for nutrition (GFN) [6], that can separate nutrient–nutrient and nutrient–non-nutrient interactions and map response surfaces for different traits (from molecular to life-history responses) in *n*-dimensional nutrient space. Adding to this explanatory power is our ability to readily measure a myriad of “intermediary” phenotypes as produced from large-scale “omics” experiments. These outcomes generate insights into how experimental factors interact to determine health. The challenge now is how best to analyze the datasets produced from these multifactorial experiments, where the number of omics features tends to be much larger than the sample size [5, 7].

A common strategy to address this challenge is to group the high-dimensional omics features into highly correlated clusters and then analyze the relationship between these clusters and experimental factors. Examples of this approach are weighted correlation network analysis [8] and ClustOfVar [9], which use unsupervised clustering of omics features based on correlation structure or their abundance value. Such methods have been widely used to analyze genomics and proteomics data [10]. However, in the case of a multifactorial nutritional experiment, these unsupervised clustering methods do not account for the experimental structure; therefore, resulting clusters could be confounded with the study design. An illustrative example of this problem is as follows (shown in Fig. S1). Consider the case where the abundance of two proteomic features, A and B, respond differently to the nutrient exposure in the presence of Drug 1 versus Drug 2. Despite responding differently to the experimental design, the marginal Spearman correlation of the two features can still be high (e.g. 0.74 in Fig. S1). As a consequence, the majority of unsupervised learning algorithms would readily group these proteins together. A further complication of using unsupervised clustering methods in the context of experimental nutrition science is that they do not provide a biological interpretation of the resulting clusters, which makes it hard to understand how experimental factors affect the responses to feature clusters [11].

We propose a novel statistical workflow for an experiment-guided NutriOmics DAta cLustering framework, which we coin eNODAL. This eNODAL workflow first uses an ANOVA-like model to distinguish whether an omics feature (e.g. a protein) shows significant response to the experimental design such as additive effects of a nutritional intervention (e.g. dietary carbohydrate) and some other external factors (e.g. drug exposure, genetic manipulation) or their interaction. Subsequently, a consensus clustering method is performed to further identify subclusters of features with similar response profiles. Finally, these subclusters are annotated based on both experimental response and pathway enrichment. This hybrid framework aims to capture both the effects of experimental treatments and similarities in the profiles of molecular features. Using data from a recent multidiet GFN study in mice [12], we demonstrate how eNODAL clusters proteomics features based on their response to an experiment involving drug–diet interactions and can then link these features to key phenotypes related to metabolic health. Using eNODAL, we identify 29 interpretable proteomics subclusters representing different responses to nutrient intake, drug exposure, and their interaction (i.e. proteins whose response to nutrient intake was substantially altered by drug exposure). Demonstrating the power of eNODAL, one such interactive subcluster, comprising proteins that are involved in the key activated protein kinase (AMPK) pathway, would not have been identified via ANOVA or correlation-based clustering methods alone.

## Material and methods

### Data

The data used come from an experimental study on the interactive effects of dietary macronutrients and gerotherapeutic drugs in mice [12]. In summary, male C57BL/6 J mice were kept on 1 of 10 different diets. The diets were designed to span across multidimensional nutrient space (protein, carbohydrate, fat, energy density), using the GFN. Each diet comprised one of five different ratios of macronutrients (i.e. % energy from protein, carbohydrate, and fat) and was replicated at two energy densities (8 and 14.8 kJ/g), with cellulose being used as the indigestible and bulking agent to control energy density. Layered over this multidimensional nutritional design, animals were also on a control (no-drug) treatment or one of three gerotherapeutic: metformin, rapamycin, or resveratrol. Thus, the experimental design included 10 diets (five macronutrient ratios across two energy densities) and four treatment groups (control, metformin, rapamycin, and resveratrol). Key metabolic traits, food intake, and the intake of individual macronutrients were measured, and the abundance of the liver proteome was quantified. The variables involved are shown in Table 1.

**Table 1:** Description of the variables in the experiments

Variable	Notation	Description
Nutrition features	W	A matrix of nutrition intake includes four columns of continuous variables: protein, carbohydrate, fat, and energy intake.
Treatment	D	A discrete variable of four levels of drug treatment: control, metformin, rapamycin, and resveratrol
Proteomics feature	Z	A matrix of proteomics measurements for each mouse with 4987 columns of continuous variables where each column represents a protein.

### Experiment-guided nutriomics data clustering method

**Figure 1:**
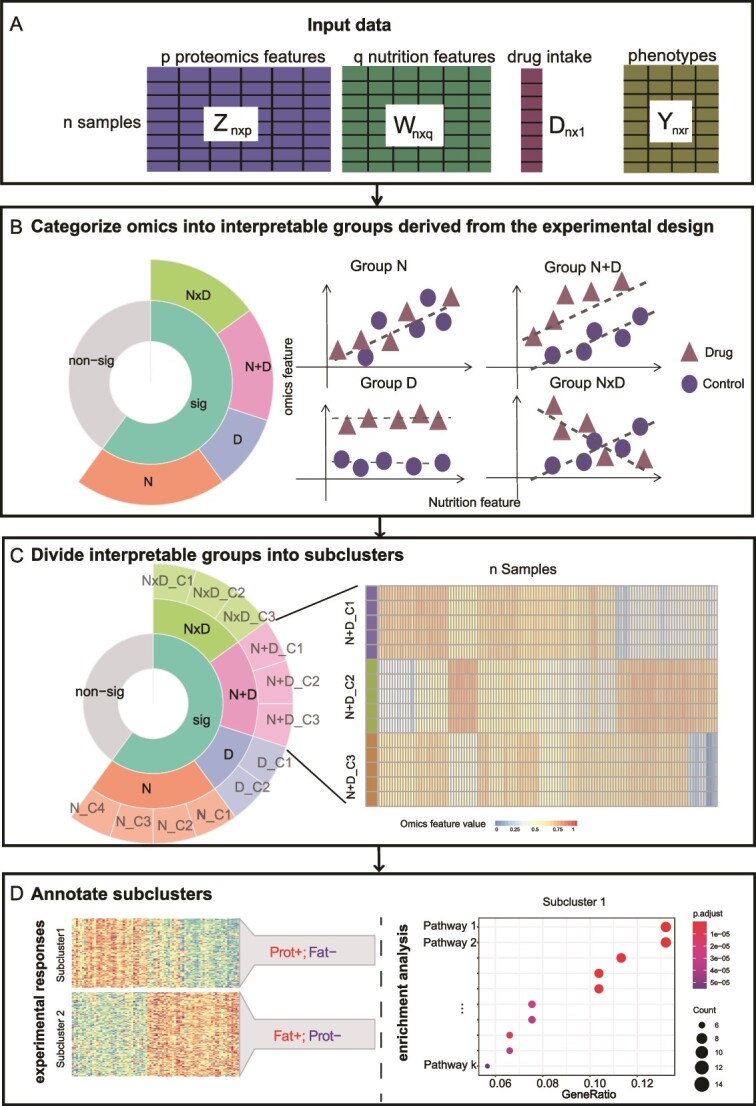
A schematic workflow for eNODAL showing four different stages. (a) Input of eNODAL including nutrition data, drug intake, omics data and metabolic phenotypes; (b) categorize omics features into interpretable groups derived from the experimental design by ANOVA-like test; (c) divide interpretable groups into subclusters via ensemble clustering; and (d) annotate subclusters based on their experimental responses and pathway enrichment analysis.

eNODAL hierarchically groups high-dimensional omics features guided by experimental factors (Fig. 1 and Supplementary Fig. S2 available online at http://bib.oxfordjournals.org/). eNODAL has three key steps: an ANOVA-like test categorizes omics features into interpretable groups based on significant effects of treatments and/or their interactions (section ANOVA-like Test). Second, a consensus clustering method further divides these interpretable groups into subclusters to reflect distinct patterns of omics features (section Consensus Clustering). Finally, these subclusters of features are annotated in two ways: [1] experimental responses (section Annotate Subclusters by Interpretable  Features), and [2] pathway enrichment (section Annotate   Subclusters by Pathway Enrichment Analysis).

### Two-stage clustering

The eNODAL framework uses a two-stage clustering method to group the high-dimensional omics features into subclusters. We will describe the details of each stage in the following sections.

#### ANOVA-like test

The development of the first step ANOVA-like test is inspired by a nonparametric ANOVA method, which was first proposed to classify genes into different groups based on their factor effect [13]. We extend this method to categorize the proteomics features based on their response to a group of continuous variables (nutrition features) and a four-level categorical factor (drug treatment). We further consider the relationships among nutrition (continuous variables), treatment (four-level categorical variable), and proteomics features (continuous variables). The nonlinear version can be found in Supplementary Notes. We define the five nested models (*M1*, *M2*, …, *M5*) as follows:


$$ M1:{z}_{ijk}={\mu}_j+{w}_i{\beta}_j+{\alpha}_{jk}+{w}_i{\gamma}_{jk}+{\epsilon}_{ijk}, $$

$$\kern-2.3pc M2:{z}_{ijk}={\mu}_j+{w}_i{\beta}_j+{\alpha}_{jk}+{\epsilon}_{ijk}, $$

$$ \kern-4.3pcM3:{z}_{ijk}={\mu}_j+{\alpha}_{jk}+{\epsilon}_{ijk}, $$

$$\kern-3.9pc M4:{z}_{ijk}={\mu}_j+{w}_i{\beta}_j+{\epsilon}_{ijk}, $$

$$\kern-5.9pc M5:{z}_{ijk}={\mu}_j+{\epsilon}_{ijk}, $$

where ${z}_{ijk}$ is the ${j}^{th}$ proteomics feature for the ${i}^{th}$ sample that received the ${k}^{th}$ gerotherapeutic drugs ($k=1$ represents the control group); $\mu$ is the overall effect; ${\alpha}_{jk}$ is the ${k}^{th}$ treatment effect on ${j}^{th}$ protein (in the mouse nutrition study, we have four different treatments corresponding to different drug intake); ${w}_i$ is the nutrition features of ${i}^{th}$ sample; and ${\beta}_j$ and ${\gamma}_{jk}$ are the effect size of the relationships between nutrition and proteomics features. For $M1$, ${\beta}_j$ and ${\gamma}_{jk}$ are used to account for the main effects of treatment and nutrition as well as their interaction. For *M2*, ${\beta}_j$ represents the contribution of nutrition intake. Proteins of $M2$ are affected by both nutrition and drugs, but their effects are independent.

Next, we categorize all proteins into five interpretable groups. Each group corresponds to one of the above five nested models, that is, $M1,M2,\dots, M5$, based on ANOVA-like testing as described below. We denote the set of all proteomics features as $S$.

The ANOVA-like testing proceeds as follows:

(1) First, we identify proteins whose abundance is affected by either nutrition or treatment. This is achieved by the Local Consistency (LC) test [14, 15], which tests whether the effect of nutrition and treatment is significantly different from randomly permuted protein abundance. This is corresponding to test *M5* (${\mathrm{H}}_0:{\alpha}_{jk}={\beta}_j={\gamma}_{jk}=0,\forall \mathrm{j},\mathrm{k}$) versus *M1*(${\mathrm{H}}_1:$ at least one parameter not equal to zero). Features that show a significant response are assigned to the cluster “sig,” denoted as ${C}_0$, and otherwise are assigned to the cluster “nonsig” ($S\backslash{C}_0$).
(2) For the proteins in cluster “sig,” we use a nested ANOVA test to test whether the interaction effect in $M1$ is significant. That is, we test for each proteomics feature, ${H}_0$: ${\gamma}_{j1}={\gamma}_{j2}={\gamma}_{j3}={\gamma}_{j4}=0$, which corresponds to *M2* versus ${H}_1$: at least one ${\gamma}_{jk}$ is not equal to zero (*M1*). The set of proteins with a significant interaction effect is denoted as ${C}_{int}\subset{C}_0$.
(3) For the proteins in the set ${C}_0\backslash{C}_{int}$, we fit $M3$ and $M4$ to test whether coefficients ${\alpha}_{jk}$ and ${\beta}_j$ is significant, which is corresponds to test for each *j*, ${H}_0$: ${\alpha}_{jk}=0\forall \mathrm{k}$, ($M4$) versus ${H}_1$: at least one ${\alpha}_{jk}$ ≠0 ($M2$), and for each *j*, ${H}_0$: ${\beta}_j$ = 0 ($M3$) versus ${H}_1$: ${\beta}_j$ ≠ 0 ($M2$), respectively. Such a test also can be done via nested ANOVA tests. Proteins with ${\alpha}_{jk}$ ≠ 0 and ${\beta}_j$ = 0 are classified as cluster “D,” denoted as ${C}_{\mathrm{D}}$, and those with ${\alpha}_{jk}$ = 0 and ${\beta}_j$ ≠ 0 are classified as group “N,” denoted as ${C}_N$.
(4) All proteins in ${C}_0\backslash \left({C}_{int}\cup{C}_N\cup{C}_{\mathrm{D}}\right)$ form the “N + D” group.

After fitting the models and calculating the *P*-value, we use a hierarchical *P*-value adjustment to correct the *P*-value. Then, the Bonferroni method is used to control the false discovery rate. Through this procedure, we classify the proteins into five interpretable groups, i.e. “N × D” (*M1*), “N + D” (*M2*), “N” (*M3*), “D” (*M4*), and “nonsig” (*M5*). A summary of the comparison, null hypothesis, and test statistics for each step can be found in Table S2.

#### Consensus clustering

Based on the categorized five interpretable groups, we further divide the groups into subclusters using unsupervised clustering methods. We use a consensus clustering method with different types of distance measurements (Supplementary Notes, Section 4 and Fig. S3) and varieties of clustering methods including affinity propagation [16], Louvain clustering based on the *k*-nearest neighbor graph [17], a dynamic tree cut method for hierarchical clustering [18], and density-based spatial clustering of applications with noise [19]. These methods use a data-driven way to find the number of clusters and adapt well to the complexity of individual datasets.

Then, a consensus matrix is created based on each individual clustering result. A binary similarity matrix is constructed from the corresponding clustering labels: if two features belong to the same cluster, their similarity is 1; otherwise, their similarity is 0. Finally, the resulting consensus matrix is clustered using the Louvain algorithm to get the resulting subclusters for each interpretable group.

### Subcluster annotation

After two-stage clustering, we annotate these subclusters from two perspectives: first, from three sets of interpretable features described in the section Calculate Interpretable Features for Each  Protein, and second, from pathway enrichment analysis. The details of the annotations of each subcluster are presented below.

#### Calculate interpretable features for each protein

Let ${z}_{ijk}$ denote the ${j}^{th}$ proteomics measurement in the ${k}^{th}$ drug treatment group ($k=1,\dots, 4$, where $k=1$ represents the control group), the corresponding ${l}^{th}$ nutrition intake is denoted ${w}_{lk}$, and ${z}_j=\left({z}_{j1},{z}_{j2},{z}_{j3},{z}_{j4}\right)$,${w}_l=\left({w}_{l1},{w}_{l2},{w}_{l3},{w}_{l4}\right)$. In the mouse nutrition study, we focus on four nutrition intake features $l=1,2,3,4$, i.e. raw food intake in grams, and protein, carbohydrate, and fat intake in kilojoules. Two sets of interpretable features for the ${j}^{th}$ proteomics measurements are described in the following.

Set 1: We first calculate the Fisher’s *z*-test statistic, ${Z}_{jl}\ (l=1,2,3,4)$ from the correlation coefficients of the proteomics feature ${z}_j$ and nutrition feature ${w}_l$:


$$ {\mathcal{Z}}_{\mathrm{j}l}=\frac{1}{2}\ln \left[\frac{1+\mathrm{cor}\left({z}_j,{w}_l\right)}{1-\mathrm{cor}\left({z}_j,{w}_l\right)}\right], $$

where $\mathrm{cor}\left({z}_j,{w}_l\right)$ is the sample correlation coefficient between protein ${z}_j$ and nutrition ${w}_l$. Each interpretable feature in Set 1 is calculated by $\frac{1}{\sqrt{n-3}}{\mathcal{Z}}_{\mathrm{j}l}$ where $n$ is the number of observations.

Set 2: We then calculate the pairwise *t*-statistic of differential abundance of ${z}_{jk}$ between control $\left(k=1\right)$ and each treatment group $\left(k=2,3,4\right)$:


$$ {\mathcal{T}}_{\mathrm{j}\mathrm{k}}=\frac{{\overline{\mathrm{z}}}_{\mathrm{j}\mathrm{k}}-{\overline{\mathrm{z}}}_{\mathrm{j}1}}{\sqrt{{\mathrm{s}}_{\mathrm{j}\mathrm{k}}^2/{\mathrm{n}}_{\mathrm{k}}+{\mathrm{s}}_{\mathrm{j}1}^2/{\mathrm{n}}_1}}, $$

where ${\bar{z}}_{jk}$ and ${\bar{z}}_{j1}$ are the sample mean of protein abundance in drug group $k$ and the control group, respectively, and ${s}_{jk}$, ${s}_{j1}$ and ${n}_k$, ${n}_1$ are the corresponding sample standard deviation and sample size respectively. Set 2 is composed of ${\mathcal{T}}_{\mathrm{jk}}$, $k=2,3,4$.

Set 1 shows the overall relationship between proteomics features and nutrition features.

Set 2 describes how liver protein abundance marginally changes with respect to different drugs.

#### Annotate subclusters by interpretable features

The three created sets of interpretable features reflect different aspects of the relationship between proteomics features and experimental factors. We further annotate each subcluster based on these features. For the *J* subcluster, we take the annotation of its interaction effect, as an example: we first transform the related interpretable features ${\tilde{Z}}_{jlk}\left(j\in J\right)$ to ${\mathcal{F}}_{jlk}$ as follows:


$$ {\mathcal{F}}_{\mathrm{j}l\mathrm{k}}=\left\{\begin{array}{l}1,\kern7.75em {\tilde{\mathcal{Z}}}_{\mathrm{j} lk}>\Phi (0.95)\\{}0,\kern1em -\Phi (0.95)\le{\tilde{\mathcal{Z}}}_{\mathrm{j} lk}\le \Phi (0.95)\\{}-1,\kern6.25em {\tilde{\mathcal{Z}}}_{\mathrm{j} lk}<-\Phi (0.95)\end{array}\right., $$

where $\Phi \left(\cdot \right)$ is the cumulative distribution function of the standard normal distribution and $\Phi (0.95)\approx 1.68$. Then we calculate the proportion of ${\mathcal{F}}_{\mathrm{j}l\mathrm{k}}=1$ or ${\mathcal{F}}_{\mathrm{j}l\mathrm{k}}=-1$ for proteins within subcluster *J*, i.e. ${P}_{Jl\mathrm{k}}=\frac{1}{\mid J\mid}\sum_{j\in J}\mathbf{1}\left({\mathcal{F}}_{\mathrm{j}l\mathrm{k}}=1\right)$ or ${N}_{Jl\mathrm{k}}=\frac{1}{\mid J\mid}\sum_{j\in J}\mathbf{1}\left({\mathcal{F}}_{\mathrm{j}l\mathrm{k}}=-1\right)$, where $\mathbf{1}\left(\cdot \right)$ is the indicator function. If ${P}_{Jl\mathrm{k}}>0.7$, it indicates that at least 70% of proteins in subcluster *J* show a significantly increased correlation with the nutrition variable $l$ in the drug group $k$ compared with the correlation coefficients in the control group. Then, we annotate cluster *J* with “increased correlation with variable $l$ in the drug group $k$.” A similar annotation procedure works for ${N}_{Jkl}>0.7$.

#### Annotate subclusters by pathway enrichment analysis

On the other hand, we also annotate each subcluster based on the enrichment of pathways in this cluster. This is done by enrichment analysis with the R package clusterProfiler [20], and the top enriched Kyoto Encyclopedia of Genes and Genomes (KEGG) pathway is also used to describe each subcluster.

### Creating the network among proteins, subclusters, and phenotypes

We first calculate the Spearman correlations between the ${j}^{th}$ proteomics feature and the ${m}^{th}$metabolic phenotype, denoted as ${\rho}_{jm}$. The *P*-value for testing against ${H}_0:{\rho}_{jm}=0$ is calculated. If the *P*-value is smaller than .01, the corresponding Spearman correlation is set to zero. Then we use a gene set enrichment analysis like the multiset test method [21] to determine the significance of the correlation between a subcluster and selected metabolic phenotype. If the *P*-value is smaller than .01, we put an edge to emphasize the link between the corresponding subcluster and phenotype. Proteomics features and subclusters are linked by proteins showing high correlation (rank top 5) with the first principal component of proteins in the subclusters. The resulting network is drawn using the R-package ggnetwork [22].

## Results

### Categorizing omics data into interpretable groups derived from experiments

**Figure 2:**
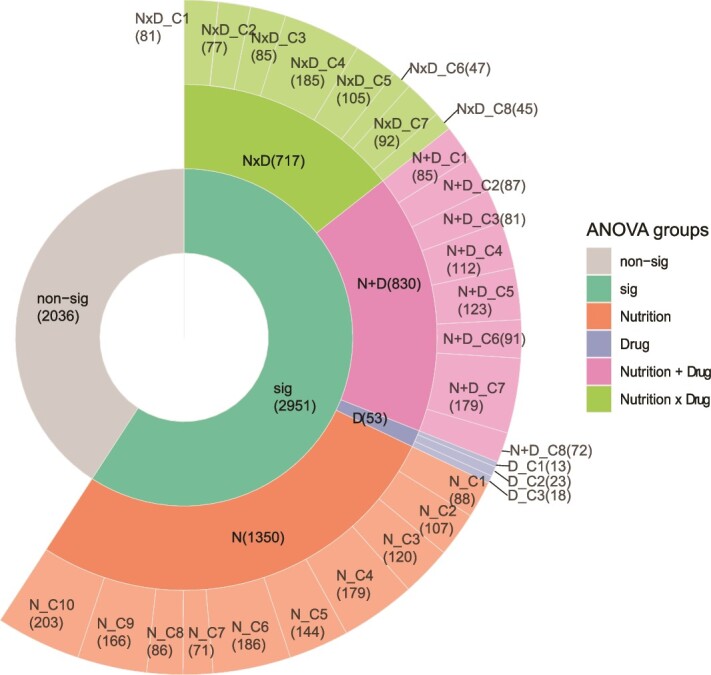
Clustering result of proteomics features from eNODAL. The 4987 proteins are categorized into four interpretable groups based on an ANOVA-like test (two inner layers). Then, it is further clustered into 29 subclusters within each group (outer layer). The numbers in each subcluster are shown within the round brackets.

In the first step of eNODAL, we categorized the high-dimensional proteomics features into interpretable groups based on whether they are significantly affected by diet, drug, and/or interactions, with the results shown in Fig. 2. A total of 2951 proteins out of 4987 proteins show significant responses to nutrient and/or drug exposure. Among these proteins with significant responses, the “N,” “N + D,” and “N × D” groups are the majority groups with 1350, 830, and 717 proteins, respectively, whereas the “D” group only has 53 proteins. The unbalanced number in each interpretable group implies nutrition shapes the largest fraction of the proteome. In contrast, a small number of proteins are affected solely by drug treatment (group “D”). That is not to say drugs have little effect on the proteome; rather, those effects occur either additively, or in a more complex interaction, with diet (i.e. in groups “N + D” and “N × D”). Pathway analysis (Fig. S4) shows that RNA splicing pathways are enriched in group “N” (Rank 1, *P* < 0.01) and “N + D” (Pank 2, *P* < .01), a finding consistent with our previous results [12]. For group “N × D,” the top enriched pathways are thermogenesis (*P* < .01) and carbon metabolism (*P* < .01). Several studies showed that thermogenesis is closely related to diet [23] and drug treatment [24]. Further, there is evidence suggesting that the interaction between drug and diet impacts thermogenesis [25]. This implies that eNODAL can group proteomics features based on their response to experimental factors.

### Dividing interpretable groups into subclusters reveals different patterns of the proteomics features

**Figure 3:**
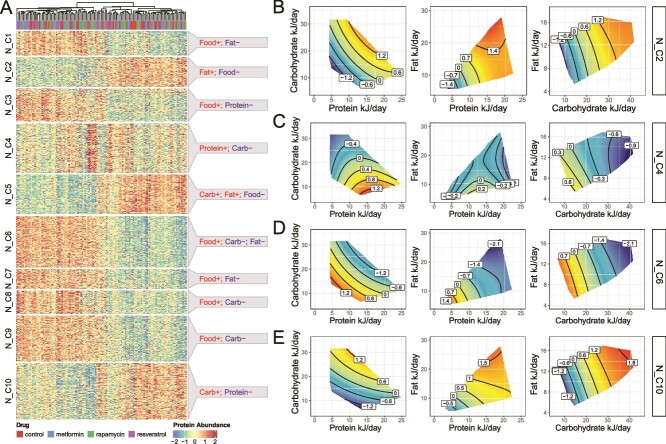
Main effect (*N* clusters): subclusters in the “N” group and their annotations. (a) Heatmap of the abundance of proteins in the “N” group, split by subclusters and annotation of each subcluster. (b–e) GFNs of the first PC of four subclusters in the ”N” group. GFN of PC1 of subclusters “N_C2,” “N_C4,” “N_C6,” and “N_C10,” respectively.

To further identify clusters of proteins with similar patterns, the consensus clustering step of eNODAL subdivided the four broad groups of experimental responses. For the “N” group, we obtain 10 subclusters. Figure 3 shows that these subclusters all have contrasting correlations with the different nutritional dimensions in the experiment (Fig. 3a). For example, Subcluster 5 in the “N” group (“N_C5”) comprises 144 proteins, the majority of which negatively correlate with total food intake in grams but positively correlate with carbohydrate and fat intake in kilojoules. Pathway analysis indicates that the peroxisome pathway, which is known to be related to lipid metabolism [12, 26], is enriched in this subcluster of proteins. To visualize the effects of nutrient intake on within-subcluster protein abundance, we apply the surfaces-based approach from the GFN to the first principal component (PC1) of abundance within each cluster (Fig. 3b–e). The subcluster “N_C5” (Fig. S5), for example, contains proteins with a higher abundance of elevated carbohydrate or protein intake, while the opposing pattern is seen in the subcluster “N_C6.” Similar results can be found within the much smaller “D” group, which is further clustered into three subclusters with different responses to drug treatment (Fig. S6).

### eNODAL reveals complex interplay among diet, drug, and metabolic pathway

**Figure 4:**
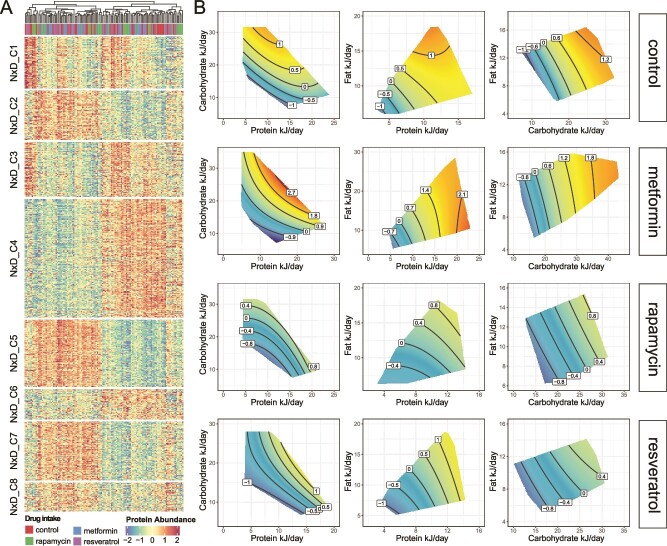
Interaction effect (“N × D” clusters): Subclusters in the “N × D” group and their annotations. (a) Heatmap of the abundance of proteins in the “N × D” group, split by subclusters. (b) GFNs of the first PC of subcluster “NxD_C4.” GFNs are fitted based on the samples for each drug treatment, respectively, resulting in 3 (combinations of nutrients intake) × 4 (number of treatments) = 12 GFNs.

Both the “N + D” and the “N × D″ group contain eight subclusters (Fig. 4a andS7). In the “N + D” group, the effects of nutrition intake and drug treatment are “additive” (as denoted by the “+” sign). Here, a combination of GFN surfaces and boxplots can be used to visualize associations between nutrition intake as well as drug treatment and the proteomics features in the cluster (Fig. S7).

For the “N × D” group, the interaction effect between nutrition and drug contributes to the abundance of proteins in these subclusters (i.e. the association between drug and protein abundance is dependent on nutrient intake). For proteins in the “N × D” groups, the interpretation of the effects of the drug needs to be evaluated with respect to the nutritional context. We visualize the association between nutrient intake and within-cluster protein abundance (based on PC1 for the cluster) using the GFN surfaces visualized for each drug group separately (e.g. Fig. 4b). Subcluster 4 in the “N × D” group (“N × D_C4”) contains the largest number of liver proteins (see Fig. 4a). For “N × D_C4” proteins, increasing energy intake leads to an elevated abundance of protein, but the presence of rapamycin and resveratrol dampens this response. In the meantime, we also observed an effect related to the protein–carbohydrate ratio (P:C) in the control group, say when energy intake is constant, increasing P:C tends to reduce the abundance of these proteins. While in drug groups, such P:C effect essentially disappears. We also see that the AMPK, insulin, and glucagon signaling pathways are enriched in this subcluster (see Fig. S8). This result is consistent with previous studies where the activation of AMPK, a nutrient-sensing pathway, has been related to the intake of metformin [27] as well as interactions between diet and metformin [28, 29].

### Network analysis reveals interplay among hub protein, subclusters, and metabolic phenotypes

**Figure 5:**
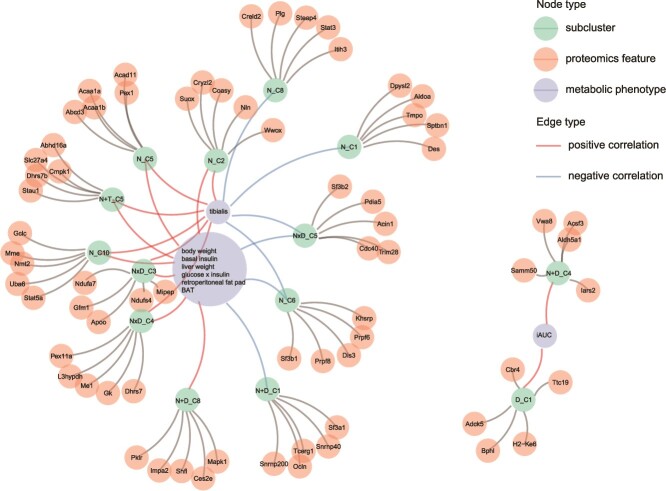
Network among hub proteins, subclusters, and metabolic phenotypes: nodes represent subclusters, hub proteins, and metabolic phenotype, respectively. Edges between subclusters and proteins determined by top five proteins correlated with the first principal component of the subcluster. Edges between subclusters and metabolic phenotypes are determined by a multiset test. Positive and negative correlations are calculated based on the correlation between the median of the subcluster and metabolic phenotype.

We jointly examined relationships between proteomics features, subclusters, and diet-related metabolic phenotypes. This step directly addresses our aim of understanding how diet by drug-affected proteins contributes to the metabolic phenotype and ultimately health of mice. This was achieved by creating a network to link proteomic features and metabolic traits and using multiset tests [21] to determine the significance of any identified associations. This analysis shows, for example, that the “N × D_C4” cluster shown in Fig. 5 links closely with a large group of metabolic phenotypes that include body weight, fasting insulin, and the mass of the retroperitoneal fat pad. Several other clusters of liver proteins that are positively affected by total energy intake also link to this cluster (e.g. “N_C2,” Figs 3b–e and5). This result is consistent with previous findings [12]. A particular protein of note is Pex11, a hub protein in subcluster “N × D_C4.” Pex11 is positively correlated with many of the metabolic phenotypes (e.g. body weight and insulin levels) in our data and in previous studies [30]. Examination of the GFN-type surfaces for this specific protein mirrors those for “N × D_C4” as a whole (Figs 4b andS9), and Pex11 has been shown to be drug–diet responsive in other studies [31, 32].

We also observed that the incremental area under the curve for insulin (iAUC) is associated with two proteomics subclusters. Both subclusters contain proteins whose abundance is elevated under rapamycin exposure (“D_C1” and “N + D_C4”). Several previous studies have noted that rapamycin exposure decreases glucose tolerance in rodents [33, 34]. Complementing this literature, eNODAL has identified that rapamycin may decrease glucose tolerance (i.e. increase the iAUC) by increasing the abundance of a suite of liver proteins (Fig. 5).

### Application of eNODAL on nutrition-microbiome data reveals the association between diet intake and abundance of *Alistipes*

To assess the generalizability of eNODAL, we applied it to an additional four nutrition–microbiome studies [35–38] to jointly examine the relationship between nutrition intake, sex, and their interaction influence the abundance of gut microbiota. Details of the datasets and processing can be found in Supplementary Notes, Section 7 and Table S1. Here, the interaction can be interpreted as whether the effect of nutrition intake is different for males and females. Our focus was on the genus level to facilitate a clearer interpretation of the findings. Unlike previous analyses, the microbiome data did not follow a normal distribution. Consequently, in the first stage of eNODAL (Two-stage Clustering), we employed the nonparametric ANOVA method [13]. We pointed out that other test methods tailored for microbiome data, such as ALDEx2 [39] and ANCOM [40], could also be utilized in this stage.

The results of eNODAL are shown in Fig. S11. Contrary to findings from previous mouse proteomics studies, these microbiome data exhibited fewer associations with nutrition intake, with more genera showing no significant associations (40/138, 6/33, 57/108, and 22/125 of significant genera for each dataset, respectively). Additionally, a notable observation across all four datasets was the absence of a significant interaction effect between nutrition and sex on the microbiome, suggesting similar gut microbial responses to nutrition in males and females.

Among the significant associations identified, *Alistipes* consistently demonstrated a significant link with nutrition intake across all studies, as illustrated by the GNF of *Alistipes* in Fig. S12. Previous research has underscored the close relationship between the abundance of *Alistipes* and nutrient intake, particularly protein [41], fat [42, 43], and carbohydrate [44]. Furthermore, previous analysis also showed that *Alistipes* serves as a mediator between fat intake and body mass index [45]. These findings collectively highlight the adaptability and utility of the eNODAL framework in uncovering complex relationships between nutrition, omics data, and phenotypes.

## Discussion

We present a three-step hybrid procedure called eNODAL, which integrates experimental structure with high-dimensional “omics” features in fully factorial nutritional studies. This framework first categorizes the features into interpretable groups based on response to experimental treatments before a consensus further divides these interpretable groups into subclusters with similar abundance profiles. Finally, we annotate these subclusters based on their experimental responses as well as enrichment of biological pathways. Demonstrating the power of eNODAL, we have analyzed data from a preclinical mouse experiment testing for interactions between diet and gerotherapeutic drugs affecting metabolic health and the liver proteome. Within these data, eNODAL obtained 29 subclusters of proteomics features representing different biological pathways. A number of these subclusters validate alternative analyses of the data, such as detecting the effects of the treatments on the spliceosome [12]. Furthermore, several of our results correlate with and complement findings from other studies on the effects of diet and gerotherapeutic drugs. For example, we see a negative effect of rapamycin on glucose homeostasis and demonstrate that these changes co-occur with the effects of the drug on a cluster of specific live proteins.

When exploring an *n*-dimensional nutrition space, this flexibility is likely to be important. Several studies [14, 15] have detected associations between nutrient intake and gene expression that could be nonlinear. We have therefore also implemented a hypothesis test using nonparametric generalized additive models (GAMs) [46–50], as well as a testing procedure to decide whether the use of a nonlinear GAM significantly alters the results relative to using a linear model (see Supplementary notes, Sections 1 and 2). In our example dataset, only 2% of proteins preferred GAM to the linear model. However, in other settings where many nonlinear relationships exist, the use of GAM in the first stage is likely to be more appropriate. A further discussion about the extension of eNODAL framework can be found in Supplementary Notes, Section 8.

The results from eNODAL provided more biological insights into the complex interplay between diet, drug, hepatic proteome, and metabolic phenotype. On the one hand, eNODAL is able to identify RNA splicing pathways enriched in the “N” group, which were also found in our previous work. Furthermore, eNODAL identifies biological pathways related to interaction effects between nutrition and drugs, such as thermogenesis and AMPK pathways. Thermogenesis is closely related to the brown adipose tissue system and has shown its important role in the regulation of body temperature [51]. Different types of diet, such as a high-fat diet and/or high-protein diet, as well as the intake of drugs, may affect thermogenesis by altering metabolism [23, 52]. The AMPK pathway is also central to metabolic regulation, including energy production and storage and synthesis of fatty acids and cholesterol. The activation of AMPK pathways could be induced both by diet and drug intake [53–55]. Understanding the complex interplay among diet, drugs, as well as related metabolic pathways, can help to optimize the effects of these substances on the regulation of the body system.

**Key Points**

- eNODAL provides an interpretable framework to categorize high-dimensional omics data, incorporating experimental design.
- eNODAL leverages a two-stage clustering strategy combining nonparametric ANOVA and unsupervised machine learning methods to offer a comprehensive annotation of resulting clusters, including both experimental response and pathway information.
- eNODAL facilitates the analysis of relationships among experimental conditions, omics features, and phenotype outcomes.
- Application of eNODAL on mouse proteomics data identified subclusters significantly affected by the interaction between nutrition intake and drug treatment.

## Supplementary Material

eNODAL_supp_BIB_bbaf036

## Data Availability

eNODAL is implemented in R and the package is freely available at our Github page: https://github.com/SydneyBioX/eNODAL All nutrition data, phenotypical data, raw, and processed proteomics data can be found in eNODAL R package (https://github.com/SydneyBioX/eNODAL) by command “data (‘Proteomics_full’)*.*”
